# LFP and oscillations—what do they tell us?

**DOI:** 10.1016/j.conb.2014.05.004

**Published:** 2015-04

**Authors:** Karl J Friston, André M Bastos, Dimitris Pinotsis, Vladimir Litvak

**Affiliations:** 1 The Wellcome Trust Centre for Neuroimaging, University College London, Queen Square, London WC1N 3BG, UK; 2 Center for Neuroscience and Center for Mind and Brain, University of California-Davis, Davis, CA 95618, USA; 3 Ernst Strüngmann Institute in Cooperation with Max Planck Society, Deutschordenstraße 46, 60528 Frankfurt, Germany

## Abstract

This review surveys recent trends in the use of local field potentials—and their non-invasive counterparts—to address the principles of functional brain architectures. In particular, we treat oscillations as the (observable) signature of context-sensitive changes in synaptic efficacy that underlie coordinated dynamics and message-passing in the brain. This rich source of information is now being exploited by various procedures—like dynamic causal modelling—to test hypotheses about neuronal circuits in health and disease. Furthermore, the roles played by neuromodulatory mechanisms can be addressed directly through their effects on oscillatory phenomena. These neuromodulatory or gain control processes are central to many theories of normal brain function (e.g. attention) and the pathophysiology of several neuropsychiatric conditions (e.g. Parkinson's disease).

## Highlights

• A brief treatment of dynamic coordination in terms of predictive coding.
• Understanding synchronous message passing in terms of hierarchical predictive coding.
• Characterising cortical gain control with the dynamic causal modelling of neural fields.
• Characterising pathophysiological oscillations with dynamic causal modelling of neural masses.

**Current Opinion in Neurobiology** 2015, **31**:1–6

This review comes from a themed issue on **Brain rhythms and dynamic coordination**

Edited by **György Buzsáki** and **Walter Freeman**

For a complete overview see the Issue and the Editorial

Available online 30th July 2014

**http://dx.doi.org/10.1016/j.conb.2014.05.004**

0959-4388/© 2014 The Authors. Published by Elsevier Ltd. This is an open access article under the CC BY license (http://creativecommons.org/licenses/by/3.0/).

## Introduction

Our review comprises four sections: the first considers the central role of gain control in hierarchical message passing and predictive coding; with a special emphasis on precision, attention and sensory attenuation. The second treats oscillations and local field potentials as fingerprints that reveal asymmetries in forward and backward extrinsic connections in cortical hierarchies. This is a prescient area of research, because it has the potential to disclose the hierarchical connectome and the putative predictive coding it supports. The third section looks more closely at horizontal connections in visual cortex and how local field potentials have been used to characterise context-sensitive changes in lateral interactions—in terms of effective connectivity and its underlying (GABAergic) synaptic gain control. Finally, we consider an example of coupling between cortical and subcortical systems that speaks to the use of oscillations in characterising pathophysiology. Specifically, we look at pathological beta oscillations and their dynamic causal modelling in Parkinson's disease.

## Oscillations, precision and predictive coding

Our treatment of oscillations rests on the premise that dynamic coordination can be understood in terms of predictive coding [1–3]. Predictive coding supposes that the brain is a statistical organ, generating predictions or hypotheses about the state of the world—predictions that are tested against sensory evidence. This (Bayesian brain) perspective is potentially important because many neuropsychiatric syndromes (ranging from autism to psychosis) can be cast in terms of false inference about states of the world (or the body) that may be due to aberrant neuromodulation or gain control at the synaptic level [4,5].

**Figure 1:**
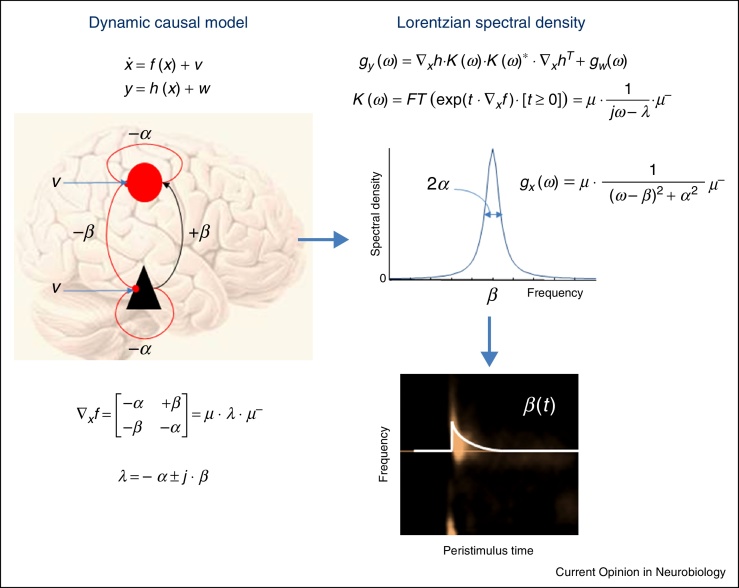
This schematic illustrates the link between the parameters of a dynamic causal model—such as effective connectivity or synaptic efficacy—and the spectral signatures of these coupling parameters. **Left panel**: state space or dynamic causal model of neuronal states *x* generating observed data *y*. The equations at the top represent the equations of motion and (static) observer function generating data. These dynamics are driven by random fluctuations *v*, where *w* represents measurement noise. The example shown here is perhaps the simplest; with recurrently and reciprocally (and linearly) coupled excitatory (black) and inhibitory (red) neuronal populations. **Right panel**: this illustrates the corresponding spectral behaviours expressed in terms of spectral densities. The top equation shows that the observed spectral density *g*(*ω*) is a mixture of signal generated by applying transfer functions *K*(*ω*) to the spectral density of the random fluctuations (assumed to be the identity matrix here for simplicity) plus a component due to measurement noise. Crucially, the transfer functions and ensuing spectral density are determined by the eigenvalues of the model's connectivity (shown on the lower left). In turn, the eigenvalues are relatively simple functions of the connectivity. The resulting (Lorentzian) spectral density is centred on the imaginary part of the eigenvalue and corresponds to the connection strength of reciprocal connections. The dispersion (full width half maximum) of the spectral peak is determined by the recurrent connectivity. This example shows how connectivity parameters can be expressed directly and intuitively in measured spectra. Furthermore, peristimulus time-dependent changes in the spectral peak disclose stimulus-induced changes in the strength of reciprocal connectivity (i.e. short-term changes in synaptic efficacy of the sort that could be mediated by NMDA receptors)—as illustrated on the lower right. In practice, dynamic causal models are much more complicated than the above example; they usually consider distributed networks of sources with multiple populations within each source and multiple states within each population—with non-linear coupling.

The circumstantial evidence for predictive coding is substantial—both in terms of the anatomy of extrinsic (between-areas) and intrinsic (within-area) connections and the physiology of synaptic interactions [3]. In these schemes, top-down predictions are used to form prediction errors at each level of cortical and subcortical hierarchies. The prediction errors are then returned to the level above to update predictions in a Bayesian sense. In brief, the prediction errors report the ‘newsworthy’ information from a lower hierarchical level that was not predicted by the higher level. A crucial aspect of this message passing is the selection of ascending information by adjusting the ‘volume’ or gain of prediction errors that compete for influence over higher levels of processing. Functionally, this gain corresponds to the expected *precision* (inverse variance or signal-to-noise ratio) that sets the confidence afforded to prediction errors. Psychologically this has been proposed as the basis of attentional gain [6]. Physiologically, precision corresponds to the postsynaptic gain or sensitivity of cells reporting prediction errors (currently thought to be large principal cells that send extrinsic efferents of a forward type, such as superficial pyramidal cells in cortex). This is important because the synaptic gain or efficacy of coupled neuronal populations determines the form of their spectral (oscillatory) behaviour. See Figure 1. Because, synchronous activity determines synaptic gain [7], oscillations have a mechanistic impact on neuronal processing—rather than being epiphenomenal—which completes the circular causality between synchrony and synaptic efficacy.

Casting hierarchical neuronal processing in terms of predictive coding has proven useful in providing formal models of behaviour and structure–function relationships in the brain. It is now arguably the dominant paradigm in cognitive neuroscience. Under predictive coding, the central role of precision—mediated by classical neuromodulatory and synchronous gain control—fits comfortably with computational and physiological formulations of neuronal processing. Crucially, electrophysiological studies of oscillations provide a rich source of empirical data for estimating synaptic efficacy. In what follows, we consider recent empirical approaches to understanding the functional architectures of predictive coding using local field potentials and dynamic causal models (DCM) of their spectral behaviour [8].

Physiologically, synaptic gain rests on a competition between excitatory and inhibitory processes. This means that a detailed characterisation of gain control should differentiate between excitatory and inhibitory postsynaptic currents. However, LFP oscillations are generated by both—creating a difficult inverse problem. DCM tries to resolve this problem with the Bayesian inversion of physiologically plausible forward models of coupled inhibitory and excitatory populations.

## Hierarchical message passing and the spectral connectome

This section focuses on recent trends in the characterisation of functional integration in cortical hierarchies. Much current work focuses on spectral asymmetries in the (functional and effective) connectivity between descending (top-down) and ascending (bottom-up) extrinsic (between area) projections. Bastos *et al.* have shown canonical patterns of *directed* interactions between different visual areas, with theta and gamma oscillations predominating in the bottom-up (or feedforward) direction and beta oscillations signal in the top-down (or feedback) direction. In addition, this metric of functional asymmetry between inter-areal oscillatory interactions predicted the underlying anatomical asymmetries in terms of laminar-specific top-down and bottom-up connections (see Vezoli, Bastos, Fries, this issue for more details). Similar spectral asymmetries in forward and backward message passing have also been found in the auditory system [9^••^]. The emerging picture is that oscillatory coupling prescribes a functional cortical hierarchy that closely matches the anatomical hierarchy [10]. The function of this hierarchy is an open question, although predictive coding models offer an intriguing explanation—in terms of hierarchical Bayesian inference.

Complementing this work, Richter *et al.* (see this issue) describe beta oscillations from extrastriate cortex to V1 in the monkey that predict the strength of evoked potentials in V1, and may therefore be a candidate mechanism for gain control. Furthermore, the top-down beta predicts stimulus-response mappings that the animal has been trained to perform. Thus, beta signals may provide top-down influences that contextualize lower-level processing. These findings fit comfortably with predictive coding models [3], which predict that top-down corticocortical connections convey prediction signals at slower time scales (e.g. beta) compared to bottom-up connections that convey prediction error signals at faster time scales (e.g. gamma). Recent work supports this hypothesis, linking fast and slow frequencies to prediction error and predictions, respectively:

Bauer and colleagues [11] have recently demonstrated that the cumulative probability of a stimulus change (a proxy for stimulus predictability) was tracked by attention-dependent alpha-band oscillations, while the inverse of cumulative probability (a proxy for surprise) was tracked by attention-dependent gamma-band oscillations. This suggests that neuronal signalling of predictions is mediated by alpha and prediction errors by gamma. These are exactly the sort of spectral dissociations one would expect if lower frequencies were involved in relaying predictions and faster frequencies in relaying prediction error.

These experimental findings are now being incorporated into models of canonical microcircuitry [3,12^••^] to understand at a mechanistic level how oscillations contribute to top-down and bottom-up processing. A key challenge for future work will be to understand not only the functional *segregation* between top-down and bottom-up signalling but also the functional *integration of these streams*, which may be subserved by laminar specific processing within the cortical microcircuit [3,13^••^].

## Gain control and lateral interactions in cortex

The preceding sections focused on the dynamic coordination among cortical areas as indexed by their spectral coupling. Here, we focus on cortical gain control (implicit in the optimisation of precision) within the intrinsic connections of the canonical cortical microcircuit. In particular, we look at recent advances in characterising excitatory-inhibitory balance—as mediated by horizontal connections within visual cortex—using dynamic causal modelling and neural fields.

Dynamic causal modelling (DCM) is a biophysically informed Bayesian framework for comparing hypotheses or network models of (neurophysiological) timeseries. It is an established procedure in the analysis of functional magnetic resonance timeseries [14,15] and is now used increasingly for the characterisation of electrophysiological measurements. There is an extensive literature on the validation of DCM ranging from face validation studies [16] to construct validation in terms of multimodal measurements [17], pharmacological manipulations [8,18] and psychophysical constructs [19]; for example, predictive coding. Predictive validity has been established in studies of pathophysiology [20]. Generally, dynamic causal modelling uses point sources (cf., equivalent current dipoles); however, recent developments now allow the use of neural fields in the forward model.

### Dynamic causal modelling of neural fields and cortical gain control

Neural fields treat neuronal signalling as a continuous process on the cortical sheet using partial differential equations [21,22]. By combining neural fields with dynamic causal modelling, one can quantify important aspects of cortical microcircuitry, like cortical excitability and the spatial reach of horizontal connections that mediate receptive field properties [23,24]. Receptive fields are not invariant to stimulus properties—their configuration is highly contrast-sensitive [25]. In [26], the authors showed that at higher contrasts, the excitatory centre of receptive fields in visual cortex (V1) had a smaller stimulus summation field, while in [27] they showed that the balance of excitatory-inhibitory influences could be modulated by stimulus context.

Neuronal responses in visual areas are sensitive to both stimulus contrast and top-down factors [28^••^]. This context-sensitivity is thought to underlie visual attention [29]. It is also known that gamma band oscillations (30–100 Hz) in V1 are sensitive to contrast, stimulus size and attention [30]. Furthermore, attention increases the peak frequency of gamma oscillations [31^••^]. This suggests an intimate link among gamma oscillations, stimulus contrast, horizontal connections and cortical gain control.

These relationships have been examined using Bayesian model comparison of dynamic causal models that embody competing hypotheses about how visual contrast effects lateral interactions [32]. Using invasive electrophysiological responses from awake-behaving monkeys several mechanisms were compared [31^••^]: candidate DCMs allowed for contrast-dependent changes in the strength of recurrent local connections [6], the strength of horizontal connections [33] or the spatial extent of horizontal connections [27]. The ability of each model to explain induced responses was evaluated in terms of their Bayesian model evidence; which provides a principled way to evaluate competing hypotheses. Bayesian model comparison suggested that increasing contrast increases the sensitivity or gain of superficial pyramidal cells to horizontal inputs from spiny stellate populations. This is consistent with precision or gain control in predictive coding—assuming that increasing contrast increases signal-to-noise. Furthermore, they provide a mechanistic explanation for why the receptive fields of V1 units shrink with increasing contrast.

## Gain control and pathophysiological oscillations

We close with an important example of dynamic coordination in pathophysiology. Namely, the emergence of pathological beta oscillations in Parkinson's disease (PD) and their characterisation with intracortical and non-invasive methods to examine the underlying directed functional connectivity (Granger causality) and effective connectivity (dynamic causal modelling).

PD is associated with degeneration of dopaminergic neurons in the substantia nigra. However, the mechanisms mediating Parkinsonian symptoms are not well understood. One robust finding—in both patients and animal models—is increased oscillations in the lower beta band (around 18–20 Hz) in the basal ganglia (BG), particularly the subthalamic nucleus (STN). Their amplitude correlates with slowness and rigidity but not tremor [34^•^]. Beta oscillations decrease with movement and their baseline level is greatly reduced by dopaminergic medication and Deep Brain Stimulation (DBS) [35]. Recent studies point to a causal role of beta in movement slowing; transcranial alternating current stimulation in the beta band slows voluntary movement in healthy subjects [36,37] and adaptive DBS triggered by high beta power appears to be superior to constant DBS in ameliorating Parkinsonian symptoms [38^••^].

The central role of abnormal beta has motivated a focus on its generative mechanisms. The reciprocally connected glutamatergic STN and GABAergic Globus Pallidus (GP) are natural candidates for generating beta [39]. A recent simulation study showed that oscillations emerge with realistic connectivity based on the latest empirical findings [40]. In light of these simulations, one might assume that beta oscillations generated in BG reach the cortex via the thalamus and ‘jam’ it. However, studies using simultaneous MEG and STN-LFP recordings in DBS patients suggest that the pathophysiology is more complicated; functional connectivity in the beta band, manifest as cortico-STN coherence, is prominent and involves ipsilateral motor areas of the cortex [41,42]. However, the frequency of this coherence does not match that of STN beta oscillations; rather it is in the upper beta band (25–30 Hz). Moreover, directed functional connectivity analyses show that the cortex drives the STN in this frequency band [41]. Additional evidence for dissociation between the pathological beta and cortico-STN coherence is the fact that the coherence is only weakly affected by dopaminergic medication and movement [43]. This cortico-STN coherence contrasts with the dopamine-sensitive synchronisation in the lower beta band—evident within and between basal ganglia nuclei, as was recently shown by looking at different cell populations within one STN [44,45] and between bilateral STN [46].

### Dynamic causal modelling of beta oscillations and synaptic gain

Dynamic causal modelling has the potential to reconcile these findings and reveal the architectures that underlie pathological oscillations. Two DCM studies of beta oscillations—one in a rat model of PD and one in patients—have been published to date [47,48^•^]. Both show an increase in cortical drive to the STN, accompanied by changes in STN-GP coupling in the pathological state. Thus DCM points to changes in synaptic gain caused by abnormal neuromodulation as the key mechanism underlying pathological increases in BG beta synchrony. How that synchrony impairs movement is still an open question—and it may transpire that the mechanism involves BG outputs to other subcortical structures, rather than disruption of motor cortical processing.

From the perspective of predictive coding, the role of beta activity fits comfortably with the observations in the visual system that top-down beta modulates the excitability of evoked responses. In the motor system, beta oscillations may reflect the precision or gain afforded by proprioceptive signals—as is evident by their attenuation during movement. This attenuation has been linked to sensory attenuation during self-made acts [49], suggesting a failure of sensory attenuation in Parkinson's disease that rests on dopaminergic modulation of beta activity [5,50].

## Conclusion

This review has considered several perspectives on how LFP oscillations can be used to inform computational and clinical models of neuronal coupling. We have focused on DCM as a way of formalising hypotheses about directed (effective) connectivity. In closing, it should be noted that—unlike descriptive (functional) connectivity measures of statistical dependencies—effective connectivity is only as good as the model that defines it. Clearly, to fully harness the macroscopic dynamics of electrophysiology, there is a long road ahead to validate current models in terms of microscopic and intracellular processes.

## Conflict of interest statement

Nothing declared.

## References and recommended reading

Papers of particular interest, published within the period of review, have been highlighted as:

• of special interest
•• of outstanding interest
